# Frontal delta-beta cross-frequency coupling in high and low social anxiety: An index of stress regulation?

**DOI:** 10.3758/s13415-018-0603-7

**Published:** 2018-05-17

**Authors:** Eefje S. Poppelaars, Anita Harrewijn, P. Michiel Westenberg, Melle J. W. van der Molen

**Affiliations:** 1 Social Psychology and Doctoral College “Imaging the Mind”, Salzburg University, Salzburg, Austria; 2 Developmental and Educational Psychology, Leiden University, Wassenaarseweg 52, 2333 AK Leiden, The Netherlands; 3 Leiden Institute for Brain and Cognition, Leiden University, Leiden, The Netherlands

**Keywords:** Amplitude-amplitude correlation, Beta, Delta, Phase-amplitude coupling, Social performance task, Social anxiety

## Abstract

Cross-frequency coupling (CFC) between frontal delta (1–4 Hz) and beta (14–30 Hz) oscillations has been suggested as a candidate neural correlate of social anxiety disorder, a disorder characterized by fear and avoidance of social and performance situations. Prior studies have used amplitude-amplitude correlation (AAC) as a CFC measure and hypothesized it as a candidate neural mechanism of affective control. However, using this metric has yielded inconsistent results regarding the direction of CFC, and the functional significance of coupling strength is uncertain. To offer a better understanding of CFC in social anxiety, we compared frontal delta-beta AAC with phase-amplitude coupling (PAC) – a mechanism for information transfer through neural circuits. Twenty high socially anxious (HSA) and 32 low socially anxious (LSA) female undergraduates participated in a social performance task (SPT). Delta-beta PAC and AAC were estimated during the resting state, as well as the anticipation and recovery conditions. Results showed significantly more AAC in LSA than HSA participants during early anticipation, as well as significant values during all conditions in LSA participants only. PAC did not distinguish between LSA and HSA participants, and instead was found to correlate with state nervousness during early anticipation, but in LSA participants only. Together, these findings are interpreted to suggest that delta-beta AAC is a plausible neurobiological index of adaptive stress regulation and can distinguish between trait high and low social anxiety during stress, while delta-beta PAC might be sensitive enough to reflect mild state anxiety in LSA participants.

**Electronic supplementary material:**

The online version of this article (10.3758/s13415-018-0603-7) contains supplementary material, which is available to authorized users.

## Introduction

Social anxiety disorder (SAD) is a debilitating disorder affecting 10.2% of adults each year and can be characterized by a persistent fear and avoidance of one or more social situations (American Psychiatric Association, 2013; Fehm, Beesdo, Jacobi, & Fiedler, 2008). SAD is associated with an increased risk to develop co-morbid disorders, especially major depression and substance use disorders (Beesdo et al., 2007; Stein et al., 2001). To increase our understanding of the etiology of SAD and aid in its early detection and prevention, it might prove useful to investigate neural correlates of social anxiety. Electroencephalography (EEG) in particular is an accessible and cost-effective method to investigate neural correlates of social anxiety, and one approach is to focus on neural oscillations, which play an important role in the large-scale synchronization of brain functions (Buzsaki & Draguhn, 2004). Cross-frequency coupling (CFC) between different neural oscillations is a key mechanism by which the brain coordinates complex cortical computations, such as information transfer and encoding (Basar, 2006; Darvas, Miller, Rao, & Ojemann, 2009), and has been studied as a candidate neural correlate of aberrant stress regulatory processes (Morillas-Romero, Tortella-Feliu, Bornas, & Putman, 2015). In particular, the amplitude-amplitude correlation (AAC) between frontal delta (1–4 Hz) and beta (14–30 Hz) oscillations seems to reflect a neural correlate of social anxiety, or even a candidate genetic trait marker (Harrewijn, Schmidt, Westenberg, Tang, & van der Molen, 2017; Harrewijn, van der Molen, van Vliet, Houwing-Duistermaat, & Westenberg, 2018). To date, however, ambiguity remains with regard to the functional significance of delta-beta AAC, both in terms of (social) stress regulation properties and biophysical properties (Canolty & Knight, 2010); this ambiguity is exacerbated by the use of between-subject measures. The current study aimed to deal with this ambiguity by comparing within-subject measures of frontal delta-beta AAC with those of phase-amplitude coupling (PAC) in social anxiety during a resting state and a social performance task.

A popular, albeit speculative, notion is that delta-beta AAC reflects the cross-talk between subcortical (delta) and cortical (beta) brain regions (Morillas-Romero et al., 2015; Schutter, Leitner, Kenemans, & van Honk, 2006). Specifically, an increase in the amplitude (or power) of delta oscillations is thought to reflect the increase of activity in a subcortical network implicated in affective processes (e.g., reward processing, impulsive and aggressive behavior, and anxiety; Knyazev, 2007), whereas an increase in beta power is thought to reflect cortically generated activity of brain regions implicated in attentional control and regulation of negative affect (Engel & Fries, 2010; Guntekin & Basar, 2010; Lopes da Silva, 1991). Increased delta-beta AAC is suggested to reflect stronger functional cortical-subcortical coherence and is associated with increased attentional control in parietal regions (Morillas-Romero et al., 2015) as well as in frontal regions (Putman, Arias-Garcia, Pantazi, & van Schie, 2012) and with reduced trait anxiety in frontal regions (Putman, 2011). This notion dovetails with fMRI findings showing that the strength of cortico-subcortical coupling between the amygdala and the frontal cortex is an index of emotion regulation efficiency (Banks, Eddy, Angstadt, Nathan, & Phan, 2007). These AAC findings seem to suggest that increased (positive) delta-beta AAC is an index of attentional control, and perhaps a plausible mechanism of regulating affective processes.

However, others have interpreted increased positive delta-beta AAC to reflect *increased* levels of anxiety in frontal areas (Miskovic et al., 2010). For example, when high (HSA) and low (LSA) socially anxious individuals were anticipating public speaking, a significant positive frontal delta-beta AAC was observed in HSA relative to LSA participants (Miskovic et al., 2010). This finding could be biased, however, by the between-subjects measure of AAC used in these studies, which not only hampers the ability to relate this measure to within-subject-based self-report traits (e.g., state/trait anxiety or social anxiety), but also limits the interpretation of its neural underpinnings within subjects. Another issue with AAC in terms of its interpretation is that it can yield negative correlations. For example, significant negative frontal delta-beta AAC was observed in HSA females relative to LSA females during the anticipation of a social performance task, as well as when recovering from this social stressor (Harrewijn, Van der Molen, & Westenberg, 2016). Such bi-directionality in coupling values can lead to delta-beta AAC values that are difficult to interpret. It has been suggested that AAC may be important for the large-scale cortical interactions that mediate cognition (Siegel, Donner, & Engel, 2012). Yet, the mechanistic and functional interpretation remains uncertain (Canolty & Knight, 2010), despite individual differences in AAC and its associations with behavior.

Besides AAC, frontal delta-beta PAC might also be implicated in stress regulation in social anxiety, and its biophysical properties are better understood. In PAC, the phase of low-frequency oscillations (e.g., delta) modulates the amplitude of high-frequency oscillations (e.g., beta), a phenomenon also referred to as “nested oscillations” (Penny, Duzel, Miller, & Ojemann, 2008; Voytek et al., 2010). PAC resembles the dynamic relationship between two different oscillations with distinct biophysical mechanisms, which ensures that the coupling strength in PAC is less sensitive to spurious coupling due to volume conduction, choice of a reference electrode, or synchronized noise (as in AAC; Dvorak & Fenton, 2014). A notion that has received increased support is that PAC offers a mechanism of neural computation and information coding, by routing the flow of information across multiple brain areas (Dvorak & Fenton, 2014; Jensen & Colgin, 2007). This concept has been validated in computational modeling studies (Chehelcheraghi, Nakatani, Steur, & van Leeuwen, 2016; Dvorak & Fenton, 2014; Sotero, 2015), as well as in human and rodent work (Engel & Fries, 2010; Van der Meij, Kahana, & Maris, 2012; Young & Eggermont, 2009). PAC has been proposed as a key neural mechanism implicated in cognitive processes such as memory formation, perception, and cognitive control (Canolty et al., 2006; Penny et al., 2008; Verguts, 2017). Together, the coupling aspects of PAC are more intuitive than AAC, since coupling is unidirectional (positive coupling only), and the biophysical properties of PAC are better understood.

However, PAC is not without its shortcomings. It has been demonstrated that the commonly used PAC method (Canolty et al., 2006) can be biased by phase-clustering that results from non-uniform phase-angle distributions, which can occur in EEG data (Van Driel, Cox, & Cohen, 2015). This phase-clustering bias can result in spurious coupling when this bias is not removed (see Van Driel et al., 2015, for a simulation study). Cox, van Driel, de Boer, and Talamini (2014) have proposed a modification to the traditional PAC method that entails a linear subtraction of the phase clustering bias from each phase angle prior to calculating PAC. The resulting debiased PAC (dPAC) effectively yields a uniform phase-angle distribution (Van Driel et al., 2015), and consequently allows for more sensitive estimations of PAC. Therefore, dPAC will be used to examine delta-beta PAC in the current study.

The purpose of this study was to investigate frontal delta-beta within-subject PAC and AAC as possible neural correlates of stress regulation in social anxiety. To induce stress, we used a social performance task (SPT; Harrewijn et al., 2016) in which participants first viewed a video-recorded presentation of a female peer, presenting her positive and negative personality characteristics. Thereafter, participants were asked to prepare a similar presentation to be videotaped and shown to other peers for evaluation afterwards. We measured EEG during anticipation of and recovery from this socially stressful situation. To capture the dynamic changes in delta-beta PAC and AAC within the anticipation and recovery conditions of the SPT, we examined the early and late stages of these two conditions. This approach could shed light on possible habituation effects (Avery & Blackford, 2016) or defensive coping (Jonas et al., 2014) that could occur over the 5-min duration of the SPT anticipation and recovery. For example, while the instruction of having to give an impromptu social performance could induce stress in both HSA and LSA participants, it is possible that LSA participants’ anxiety levels habituate over the course of the anticipation condition, while HSA participants’ anxiety levels do not habituate (potentially influenced by adaptive vs. maladaptive stress regulation strategies). In turn, this could lead to stronger delta-beta PAC or AAC in HSA participants when the stressor becomes more imminent (i.e., late anticipation condition). To examine whether delta-beta PAC is more sensitive to stress-induction (state-dependent) or reflects a more perpetual or default response to regulate stress (Brosschot, Verkuil, & Thayer, 2017), we examined delta-beta PAC and AAC during resting state as well.

Based on the ambiguity regarding the functional significance of the delta-beta AAC metric, we formulated competing hypotheses regarding the behavior of PAC and AAC in this study. If frontal delta-beta PAC and AAC was indicative of cortical-subcortical dysfunction in social anxiety (Miskovic et al., 2011), higher frontal delta-beta PAC and AAC was expected in HSA relative to LSA participants, with highest coupling in HSA participants at the later part of the anticipation condition and the early part of the recovery condition. However, if frontal delta-beta PAC and AAC was indicative of adaptive stress regulation (Putman, 2011), the reversed pattern can be expected, namely higher frontal delta-beta PAC and AAC in LSA relative to HSA participants.

## Methods

### Participants

Three hundred and eighty-six students between 18 and 25 years of age were screened for social anxiety using the Liebowitz Social Anxiety Scale (LSAS; Fresco et al., 2001; Liebowitz, 1987). Participants were recruited within the proximity of Leiden University, the Netherlands, and were invited to participate in a larger study including two paradigms: a social evaluation paradigm (reported elsewhere) and the social performance task. Participants were healthy, free from psychoactive medication, had normal or corrected-to-normal vision, and were right-handed.^1^ Since social anxiety and stress-reactivity research has shown gender-specific effects, only female participants were used for this study (Kudielka & Kirschbaum, 2005; Nolen-Hoeksema, 2012). A LSAS cut-off score of 60 provides the best balance of sensitivity and specificity for classifying participants with the generalized subtype of SAD (Mennin et al., 2002). Thus, participants were considered high socially anxious (HSA) when LSAS scores were ≥ 60. Participants were considered low socially anxious (LSA) when LSAS scores were < 30. Thirty-seven LSA and 31 HSA women were selected, corresponding to the highest 8% and lowest 9.5% of the 386 individuals of the student population who were screened with the LSAS. Sixteen participants were excluded, based on left-handedness (*n* = 3) or ambidexterity (*n* = 1) assessed with the Edinburgh handedness inventory (Oldfield, 1971); use of prescribed psychiatric medication (*n* = 2); a previous or current psychiatric/medical illness (*n* = 1); different group placement based on LSAS score between screening and testing (*n* = 2); technical difficulties (*n* = 1); or EEG artefacts (*n* = 6). Thus, the final sample consisted of 20 HSA (mean age = 19.7, *SD* = 1.5) and 32 LSA (mean age = 20.0, *SD* = 1.6) age-matched female participants.

### Procedure

Participants were invited to come to the lab based on an eligibility check of their social anxiety status (see above). Experimenters were blind to the group allocation. The procedure lasted for a total duration of 2.5 h. Participants filled in a visual analog scale to measure baseline nervousness and approach motivation (see Self-report SPT ratings section) and completed a resting state of 5 min with their eyes closed. Thereafter, participants performed a social judgment paradigm (data presented in Van der Molen, Harrewijn, & Westenberg, 2018) and the SPT. After EEG equipment was disconnected, participants filled out self-report questionnaires that were relevant to social anxiety (see Self-reported traits section). At this moment, the LSAS was administered again, to validate participants’ screening score. Finally, participants were debriefed and compensated with a monetary reward (€17) or course credit. All participants provided informed consent and the procedure was reviewed and approved by the ethics committee of the Institute of Psychology of Leiden University.

### Social performance task (SPT)

**Fig. 1:**
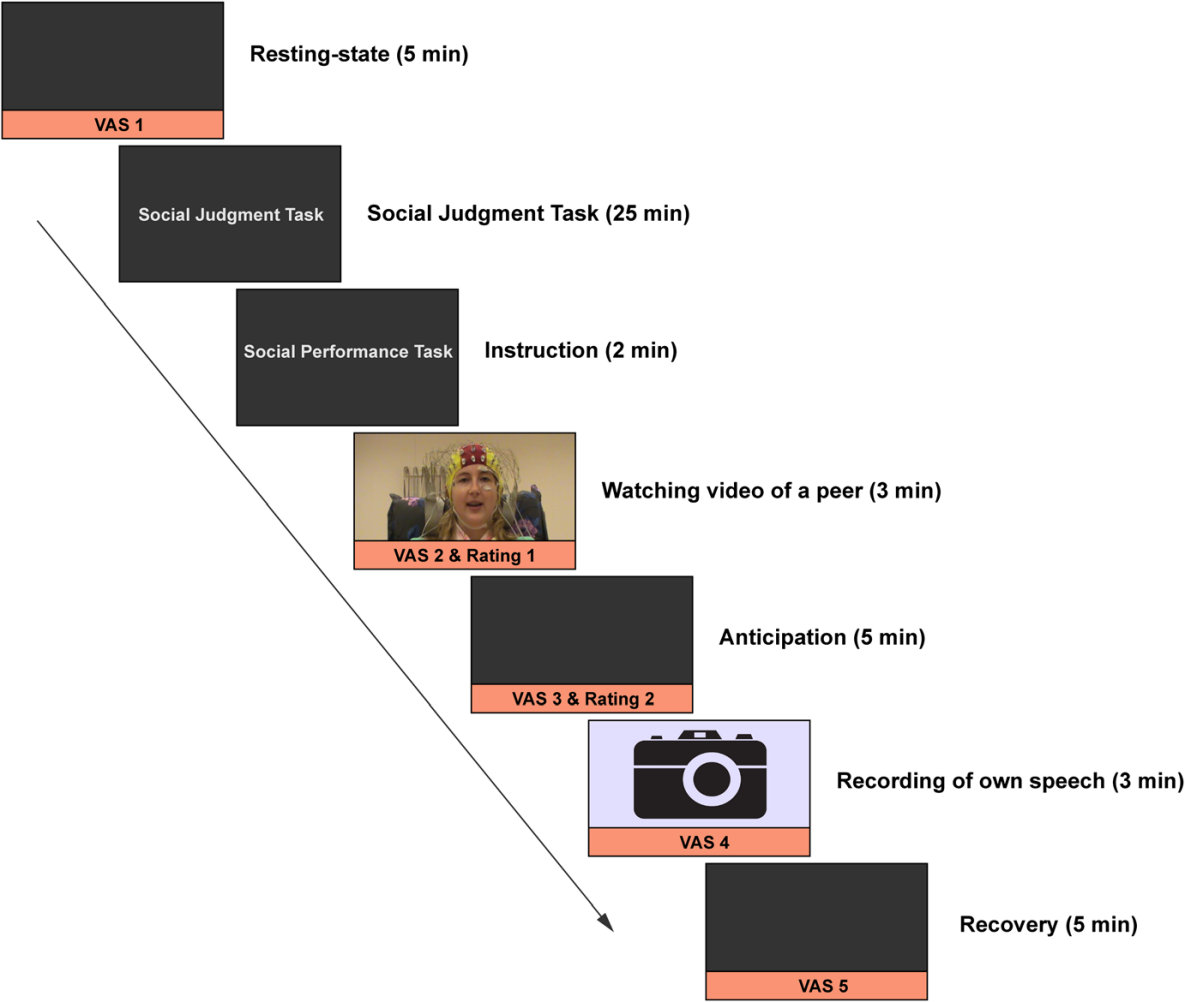
Overview of the experiment. EEG was recorded during resting state, anticipation, and recovery. The results of the social judgment task are reported elsewhere. Reprinted from Cognitive, Affective & Behavioral Neuroscience, Harrewijn, A., Van der Molen, M.J.W., & Westenberg, P.M., Putative EEG measures of social anxiety: Comparing frontal alpha asymmetry and delta-beta cross-frequency correlation, Copyright (2016), with permission

To elicit social stress, we used the SPT as described in Harrewijn et al. (2016) that comprised five conditions (instruction, video, anticipation, presentation, and recovery), which were presented in a fixed order (see Fig. 1). After the resting state and the social judgment task, participants were instructed for the first time about the upcoming SPT. This was done to warrant the impromptu character of the SPT, and to provide assurance that the resting state recording was not confounded by anticipatory stress related to the SPT. Using a cover story, participants were given an explanation that they would watch and evaluate a video of a female peer (in reality a confederate), presenting her positive and negative personality traits. Participants were then asked to record a similar video that would be evaluated by a peer at a later time (implicating social-evaluative threat). Participants then watched the 3-min self-presentation video of the peer and rated the peer on several qualities on a visual analog scale: “How socially competent/attractive/nervous is this person?” and “I would like to meet this person.” Participants also rated their nervousness and approach motivation. Then, participants prepared their self-presentation for a duration of 5 min while EEG was measured (anticipation condition). Next, participants rated how they expected that their own video would be evaluated by a peer, and again rated their nervousness and approach motivation. Participants then performed their 3-min self-presentation, revealing their negative and positive personality traits in front of a camera. Directly after their presentation, participants had 5 min to recover while EEG was again measured (recovery condition). Finally, participants rated their nervousness and approach motivation for the last time. During both the anticipation and recovery conditions, participants had their eyes open. The cover story was used to make the peer evaluation component of the SPT more convincing. In reality, the videos of the participants were not shown to anyone other than the experimenters. As stated in the exit interview, none of the participants had doubts about the cover story.

### Self-report SPT ratings

Visual analog scales were used to measure task-related nervousness and approach motivation levels, by responding to the statements “I feel nervous right now” and “I am looking forward to the next part of the experiment” (respectively) by marking a line on a scale ranging from 0 = absolutely not and 100 = absolutely. This scale was administered on five different occasions during testing (once before the resting state and four times during the SPT) and showed a satisfactory to good internal consistency in the current sample (α = .79 and .86, respectively).

### Self-reported traits

Social anxiety was measured with the Liebowitz Social Anxiety Scale (LSAS; Fresco et al., 2001; Liebowitz, 1987). This scale consists of 24 statements on four-point Likert-scales, for each of the two subscales of fear (0 = none, 3 = severe) and avoidance (0 = never, 3 = almost always). Scores range from 0 (no social anxiety) to 144 (very serious social anxiety). The LSAS was administered twice: once during screening and once during testing, and was very strongly correlated, *r* = .89, *p* < .001. It showed an excellent internal consistency in the current sample (both *α*s = .95). Participants who had a different group placement based on LSAS score between screening and testing were excluded (*n* = 2).

In addition, to validate the HSA and LSA groups on other social anxiety-related constructs (to be reported elsewhere), questionnaires were administered that measured fear of negative evaluation (Carleton, McCreary, Norton, & Asmundson, 2006; Leary, 1983), fear of positive evaluation (Weeks, Heimberg, & Rodebaugh, 2008), depression (Beck, Steer, & Brown, 1996), self-esteem (Rosenberg, 1965), post-event rumination (Edwards, Rapee, & Franklin, 2003), and cognitive emotion regulation strategies (Garnefski, Kraaij, & Spinhoven, 2001). For the cognitive emotion regulation strategies questionnaire, two scales were used: a positive (adaptive) emotion regulation scale, containing the subscales: acceptance, positive refocusing, refocus on planning, positive reappraisal, and putting into perspective; and a negative (maladaptive) emotion regulation scale, containing the subscales: self-blame, rumination, catastrophizing, and blaming others (Garnefski et al., 2001). It showed a good internal consistency in the current sample (α = .89 and α = .81, respectively). For the post-event rumination questionnaire, the negative rumination subscale was used as an index of post-threat negative rumination (Edwards et al., 2003). The instruction was adapted to address the timeframe in between the SPT and filling in the questionnaire 20 min later. Items regarding topic selection and received feedback were removed (4, 5, 7, 12, 15, 20, 28). It showed an excellent internal consistency in the current sample (α = .94).

### Signal recording and analyses

EEG was recorded continuously at a 1,024 Hz sampling rate with the ActiveTwo system (BioSemi) using 64 Ag-AgCl electrodes mounted in an elastic electrode cap (10/20 placement). The Biosemi Common Mode Sense (CMS) active electrode and Driven Right Leg (DRL) passive electrode replaced the conventional ground electrode, and CMS was used as the online reference. To monitor eye blinks and movements, horizontal EOG was measured with two Ag-AgCl electrodes placed on the left and right canthus; vertical EOG was measured with two Ag-AgCl electrodes placed above and below the left eye. Two Ag-AgCl electrodes were placed on the mastoids, and two Ag-AgCl electrodes were placed on the chest (modified lead-2 placement) to measure heart rate. Offline pre-processing of the EEG time series was performed using Brain Vision Analyzer (BVA version 2.0.4, Brain Products GmbH, 2015). The continuous EEG signal was down-sampled to 512 Hz, re-referenced to the average of all 64 electrodes and offline band-pass filtered between 0.5 and 40 Hz (24dB/oct), with a 50-Hz notch filter (zero-phase shift). Ocular artefacts were removed from the SPT data (anticipation and recovery) using the automatic ocular correction ICA method as implemented in BVA. Thereafter, for each condition, data were segmented into 8-s non-overlapping epochs (4,096 time samples), allowing for sufficient low-frequency cycles to detect dPAC (Aru et al., 2015; Cohen, 2014). The first and last ten epochs of both tasks were manually inspected for gross artifacts and excluded if necessary. Out of those, six early and six late clean epochs were randomly selected for use in further analysis.^2^ The six early and six late epochs of the resting state, anticipation, and recovery were exported to ASCII files for further analyses. Since we expected no time-related differences for the resting state, three early and three late epochs were randomly selected and combined for this condition. Our focus was on frontally mediated delta-beta AAC / PAC, to facilitate comparisons with relevant prior studies (Harrewijn et al., 2016; Miskovic et al., 2010; Putman, 2011; Putman et al., 2012), and thereby reduce the multiple-comparison problem. The electrodes of interest were a composite measure of the average of three frontal electrodes: F3, Fz, and F4, to facilitate comparisons with prior relevant studies on this topic (cf., Harrewijn et al., 2016; Putman, 2011; Putman et al., 2012). Subsequent PAC and AAC analyses were performed in MATLAB (The MathWorks, Inc., Natick, MA, USA). The selected EEG epochs were down-sampled to 128 Hz,^3^ and band-pass filtered separately for delta (1–4 Hz) and beta (14–30 Hz) using a Butterworth IIR bandpass filter by using a zero phase-shift filtering method (with a filter order of 8 for delta and 34 for beta; which doubled after using both a forward and a backward filter). A Hilbert transform was applied to the delta and beta filtered epochs to isolate the phase and amplitude information (Papoulis & Pillai, 2000). The first and last 16 samples – equal to the order of the lower frequency’s filter (cf., Knyazev, 2011) – were cut from each epoch to remove edge artefacts originating from filtering (Aru et al., 2015; Kramer, Tort, & Kopell, 2008).

### Phase-amplitude coupling analysis

PAC analyses between delta phase and beta amplitude were performed using the debiased PAC (dPAC) method (Cox et al., 2014; Van Driel et al., 2015) with custom-written scripts (freely available via https://github.com/ESPoppelaars/Cross-frequency-coupling), which were modified from Van Driel et al. (2015) to fit the current data specifications and research interests.^4^ Delta-beta dPAC and the accompanied Z-values were calculated for each participant and electrode, over the six epochs, and were thereafter averaged over the three electrodes, yielding one dPAC and Z-value per participant, per condition. dPAC was calculated by removing the phase clustering from the traditional PAC method (cf., Canolty et al., 2006) via a simple linear subtraction (cf., Cox et al., 2014; Van Driel et al., 2015). PAC can be defined as:

$$ PAC=\sum \limits_{t=0}^n{\alpha}_t{e}^{i{\varphi}_t} $$

where *a*_*t*_ denotes the amplitude of the modulated frequency (i.e., beta amplitude), and *φ*_*t*_ denotes the phase of the modulating frequency (i.e., delta phase), *t* is time, and *n* is the total number of time samples. The phase clustering (PC) is calculated by averaging the complex vector of phase angles $\left({e}^{i{\varphi}_t}\right)$, from which the magnitude (or strength) and angle of clustering can be determined:

$$ PC=\frac{1}{n}\sum \limits_{t=1}^n{e}^{i{\varphi}_t} $$

Note that by not including the beta amplitude *a*, all complex numbers have the same length, and, therefore, all angles have the same weight in the averaging process. This allows for determining the average angle, or PC. For dPAC, the aforementioned complex numbers, *a*_*t*_*e*^*iφ*^ (combining beta amplitude *a* and delta phase *φ*) are averaged for all time samples, correcting the phase angle of the complex numbers by the earlier obtained PC:

$$ dPAC=\frac{1}{n}\sum \limits_{t=1}^n{a}_t\left({e}^{i{\varphi}_t}- PC\right) $$

The dPAC value is expressed as the magnitude of the averaged complex number, where zero indicates no coupling, and values greater than zero indicate coupling. The significance of the coupling was established by comparing the dPAC values to surrogate dPAC values that were obtained via a non-parametric permutation testing approach (Maris & Oostenveld, 2007) by randomly shuffling epochs for phase information, while amplitude remained intact. This shuffling process was repeated 1,000 times, yielding a distribution of surrogate dPAC values as expected under the null hypothesis of no coupling. This method not only allows for significance testing but also accounts for possible outliers (Van Driel et al., 2015). Significant dPAC was determined by comparing dPAC to their surrogate counterparts (*dPAC*_*null*_) to obtain *Z*-values (dPACz):

$$ dPACz=\frac{dPAC- mean\left({dPAC}_{null}\right)}{std\left({dPAC}_{null}\right)} $$

These *Z*-values were used for hypothesis testing due to their straightforward interpretation (i.e., standard deviation units) (Cohen, 2014).

### Amplitude-amplitude correlation analysis

The current AAC analysis was based on the within-subject AAC analysis (Knyazev, 2011) and diverged from more common AAC analyses (Harrewijn et al., 2016) in order to be able to compare the current dPAC and AAC results. The delta and beta amplitude in the Hilbert-transformed epochs (power envelopes obtained in “Signal recording and analyses” section) were treated as two time series and correlated over all timepoints. After calculating these correlation coefficients for each participant and electrode over the six epochs, the three electrodes were averaged, yielding one correlation coefficient per participant, per condition.

### Statistical analysis

For statistical analysis, SPSS version 23 (IBM Corp., 2015) was used. Due to the non-normal distribution of self-reported traits, SPT ratings, dPAC Z-values and AAC correlation coefficients, non-parametric tests were used. Group differences were analyzed using Mann-Whitney U tests. Within-group significance of Z-values (dPAC) and correlation coefficients (AAC) were assessed using one-sample Wilcoxon signed rank tests. Correlations between EEG data (dPAC and AAC) and self-reported characteristics (LSAS, post-threat negative rumination, state nervousness, state approach motivation) were done with Spearman’s rho correlations within each group.

Multiple comparisons correction for dependent samples was done by using the false discovery rate (FDR; Benjamini & Yekutieli, 2001) for every section separately by using online resources (http://www.sdmproject.com/utilities/?show=FDR). All reported *p*-values are FDR-corrected. The significance level was set at α = 0.05.

## Results

### Self-reported traits

Table 1 present the mean scores on the self-report questionnaires from the LSA and HSA participants. As expected, HSA participants reported significantly higher social anxiety, fear of negative and positive evaluation, depression, and lower self-esteem, as compared to LSA participants. The groups did not differ on positive and negative emotion regulation strategies.

**Table 1:** Self-reported trait scores related to anxiety

	LSA mean (*SD*)	HSA mean (*SD*)	U	*Z*	*p*
Social anxiety (screening)	19.06 (7.5)	72.35 (11.6)	.00	6.02	<.001***
Social anxiety (testing)	24.91 (11.7)	76.55 (19.8)	2.50	5.97	<.001***
Fear of negative evaluation	19.59 (12.2)	29.75 (10.1)	163.50	2.95	.008**
Fear of positive evaluation	23.29 (14.0)	38.45 (11.9)	125.00	3.57	<.001***
Self-esteem	21.32 (4.2)	16.65 (4.5)	126.50	3.56	<.001***
Positive ER strategies	3.26 (.51)	3.36 (.78)	283.50	0.33	.744
Negative ER strategies	2.23 (.47)	1.98 (.47)	212.00	1.74	.103
Post-threat Neg. Rum.	14.35 (9.7)	26.95 (11.0)	117.50	3.64	<.001***
Depression	7.19 (5.1)	12.15 (8.0)	183.00	2.46	.023*

The bold indicators of significant findings are presented in the outer right column (indicated as *p* values)

False discovery rate (FDR)-corrected *p*-values are displayed

*LSA* low socially anxious group, *HSA* high socially anxious group, *ER* emotion regulation, *Neg. Rum.* negative rumination

*Significant at a FDR-corrected α-level of 0.05

**Significant at a FDR-corrected α-level of 0.01

***Significant at a FDR-corrected α-level of 0.001

### Self-report SPT ratings

**Fig. 2:**
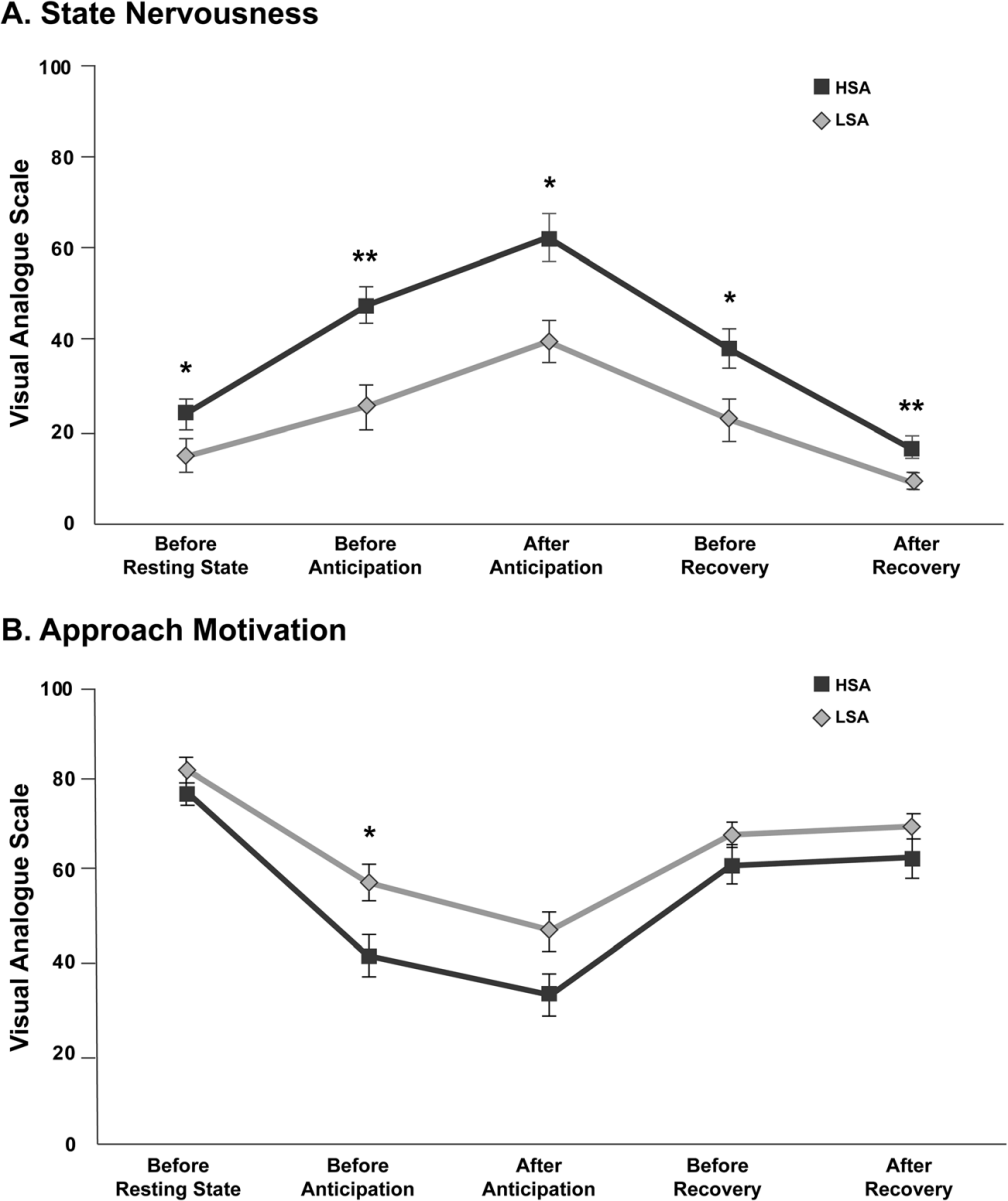
Self-report ratings of state nervousness (**panel A**) and approach motivation (**panel B**) after the resting state and SPT conditions for high socially (HSA) and low socially anxious (LSA) participants. Error bars represent standard errors of the mean. * Significant group difference at a FDR-corrected α-level of 0.05; ** Significant group difference at a FDR-corrected α-level of 0.01

Mean scores for nervousness and approach motivation per group are plotted in Fig. 2A and B. Regarding SPT ratings of nervousness and approach motivation, HSA participants reported more nervousness for both resting state and all SPT conditions than LSA participants, as well as less approach motivation before anticipation (*p*s < .05, FDR corrected).

### Frontal delta-beta debiased phase-amplitude coupling

Simulated data with and without introduced CFC were analyzed to assure proper functioning of the analysis script. Results showed that dPAC was significant in the simulated datasets both with and without introduced CFC. However, there was significantly more dPAC in the simulated data with introduced CFC. See the Supplementary Materials for further details.

#### dPAC within groups

Delta-beta dPAC results are displayed in Table 2. One-sample Wilcoxon signed rank tests showed that delta-beta dPAC Z-values differed significantly from zero for both LSA and HSA participants during the resting state, as well as the early and late anticipation, and early and late recovery conditions of the SPT (all *p*s < .05, FDR-corrected). Detailed statistics are displayed in Table 2.

**Table 2:** Delta-beta phase-amplitude magnitude and within-group statistics

Condition	dPAC: mean magnitude (SD)		dPAC: mean Z-value (SD)		Z		*p-values*	
	LSA	HSA	LSA	HSA	LSA	HSA	LSA	HSA
Resting state	.060 (.031)	.046 (.019)	.88 (.40)	.80 (.43)	4.94	3.92	<.001***	<.001***
Early anticipation	0.059 (.026)	.056 (.030)	.90 (.38)	.75 (.37)	4.94	3.92	<.001***	<.001***
Late anticipation	.055 (.031)	.050 (.019)	.87 (.42)	.80 (.28)	4.94	3.92	<.001***	<.001***
Early recovery	.050 (.025)	.057 (.035)	.84 (.35)	.86 (.32)	4.94	3.92	<.001***	<.001***
Late recovery	.052 (.027)	.049 (.025)	.93 (.46)	.78 (.39)	4.94	3.92	<.001***	<.001***

False discovery rate (FDR)-corrected *p*-values are displayed

*LSA* low socially anxious group (n=32), *HSA* high socially anxious group (n=20), *dPAC* debiased phase-amplitude coupling, *Z* Wilcoxon standardized test statistic

***Significant at a FDR-corrected α-level of 0.001

#### dPAC between groups

**Fig. 3:**
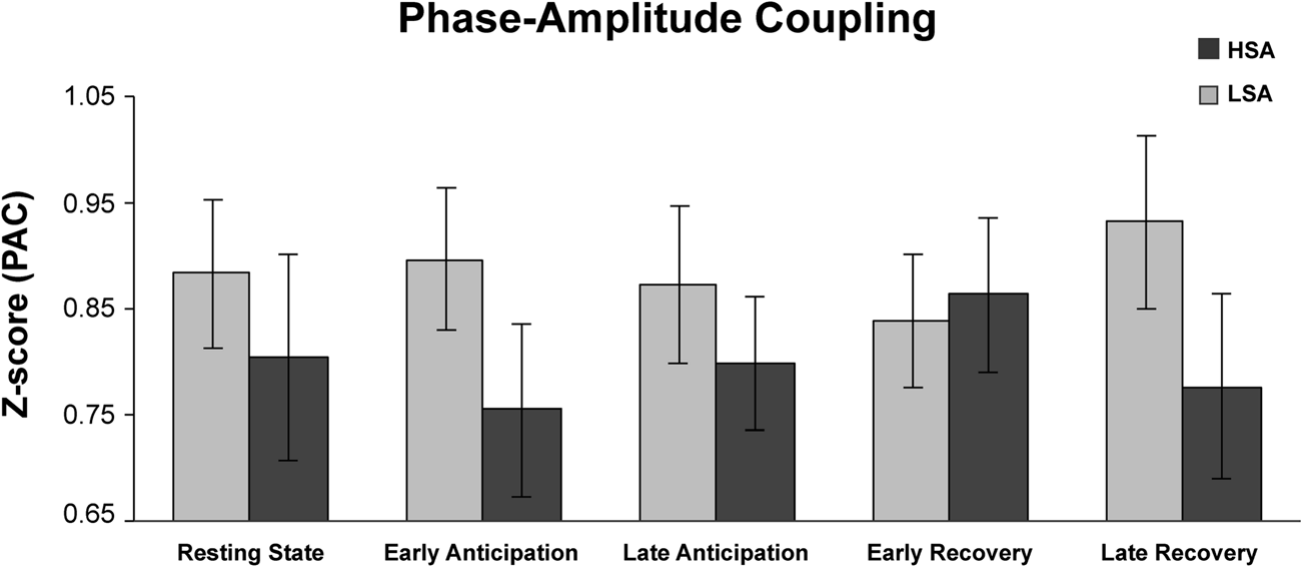
Delta-beta debiased phase-amplitude coupling results during resting state and the SPT conditions for the high socially (HSA) and low socially anxious (LSA) participants. *Note:* Error bars reflect standard errors of the mean. Group differences in dPAC did not reach levels of significance at a FDR-corrected α-level of 0.05

Delta-beta dPAC results are displayed in Fig. 3. Mann-Whitney U tests showed no differences between LSA and HSA participants in delta-beta dPAC during resting state, Z = 0.92, FDR-corrected *p* = 0.595; early anticipation, *Z* = 1.22, FDR-corrected *p* = .552; late anticipation, *Z* = .34, FDR-corrected *p* = .821; early recovery, *Z* = .22, FDR-corrected *p* = .821; or late recovery, *Z* = 1.43, FDR-corrected *p* = .552. Results are displayed in Fig. 3.

### Frontal delta-beta amplitude-amplitude correlation

Simulated data with and without introduced CFC were analyzed to assure proper functioning of the analysis script. Results showed that AAC was only significant in the simulated dataset with introduced CFC, and not in the dataset without CFC. Moreover, there was significantly more AAC in the simulated data with introduced CFC. See the Supplementary Materials for further details.

#### AAC within groups

One-sample Wilcoxon signed rank tests showed that delta-beta AAC correlation coefficients differed significantly from zero for LSA participants during the resting state, as well as the early and late anticipation, and early and late recovery conditions of the SPT (all *p*s < .05, FDR-corrected). However, the HSA group showed no significant delta-beta AAC during any of the conditions (all *p*s > .167, FDR-corrected). Detailed statistics are displayed in Table 3.

**Table 3:** Delta-beta amplitude-amplitude correlation coefficients and within-group statistics

Condition	AAC mean (SD)		*Z*		*p*-values	
	LSA	HSA	LSA	HSA	LSA	HSA
Resting state	.027 (.047)	.013 (.034)	2.71	1.31	.020*	.239
Early anticipation	.034 (.035)	.008 (.034)	3.94	1.57	<.001***	.167
Late anticipation	.018 (.039)	.007 (.034)	2.26	.37	.048*	.709
Early recovery	.031 (.049)	.007 (.039)	3.01	.48	.015*	.697
Late recovery	.021 (.038)	.023 (.051)	2.65	1.57	.020*	.167

False discovery rate (FDR)-corrected *p*-values are displayed

*LSA* low socially anxious group (n=32), *HSA* high socially anxious group (n=20), *AAC* amplitude-amplitude correlation, *df* degrees of freedom

*Significant at a FDR-corrected α-level of 0.05

**Significant at a FDR-corrected α-level of 0.01

***Significant at a FDR-corrected α-level of 0.001

#### AAC between groups

**Fig. 4:**
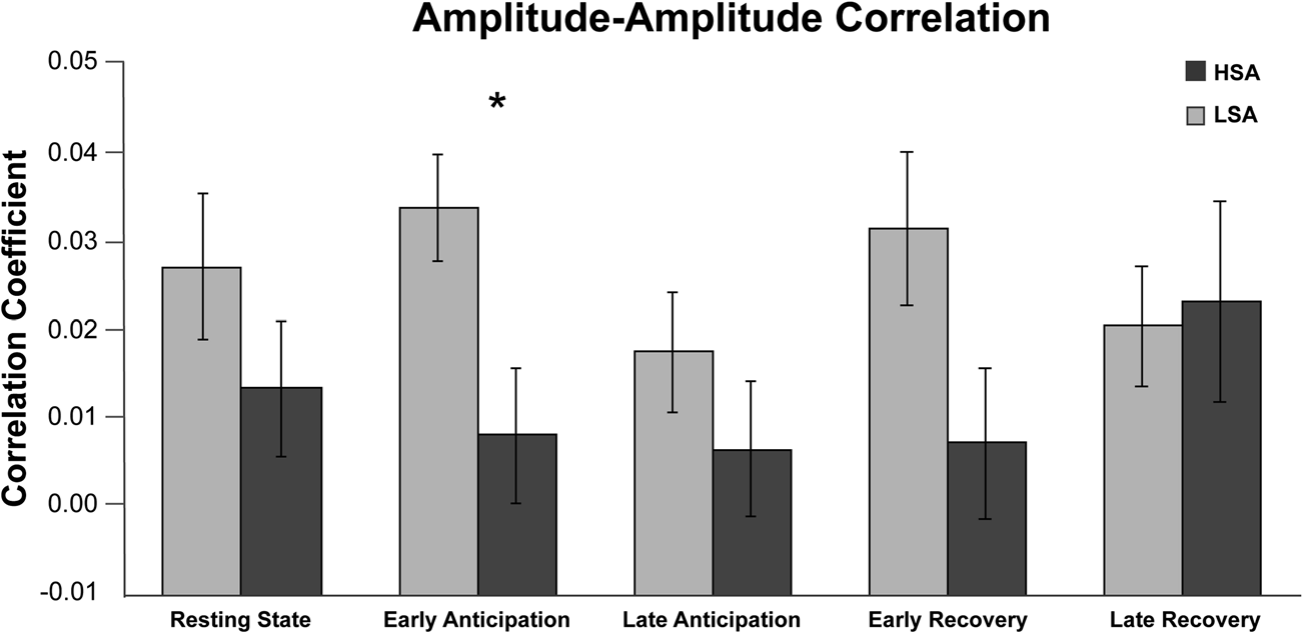
Delta-beta amplitude-amplitude correlation (AAC) during resting state and the SPT conditions for the high socially (HSA) and low socially anxious (LSA) participants. *Note:* Error bars reflect standard errors of the mean. LSA participants showed significantly more AAC than HSA participants during early anticipation at a FDR-corrected α-level of 0.05

Delta-beta AAC for the LSA and HSA groups is shown in Fig. 4. Mann-Whitney U tests showed a significant difference in delta-beta AAC between LSA and HSA participants during early anticipation, *Z* = 2.73, FDR-corrected *p* = .030, but no group differences during resting state, Z = 0.85, FDR-corrected *p* = 0.496; late anticipation, *Z* = .1.13, FDR-corrected *p* = .432; early recovery, *Z* = .1.96, FDR-corrected *p* = .125; or late recovery, *Z* = .30, FDR-corrected *p* = .763. Results are displayed in Fig. 4.

### Correlations between EEG data and self-reported traits and SPT ratings

**Fig. 5:**
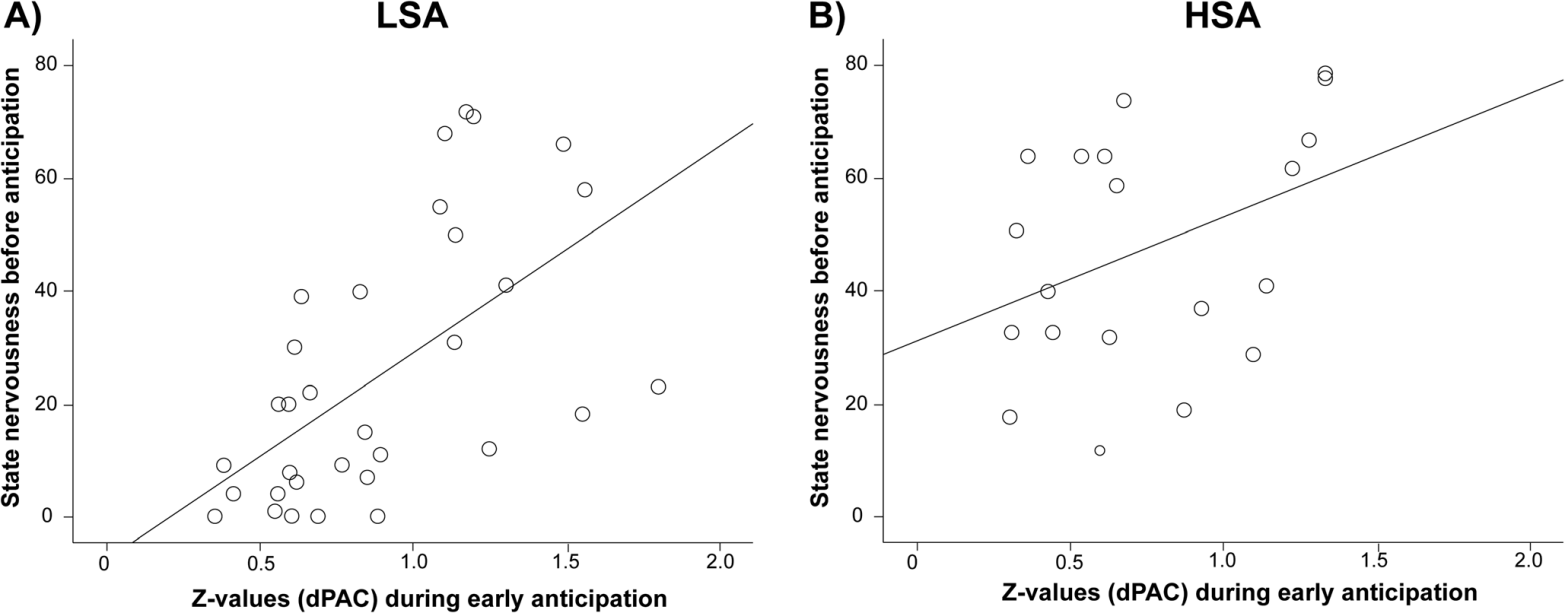
Correlation between state nervousness before anticipation and dPAC during early anticipation for (**A**) the low socially anxious (LSA) group (*rho* = .632, FDR-corrected *p* = .007), and (**B**) the high socially anxious (HSA) group (*rho* = .440, FDR-corrected p = .879)

Spearman’s rho correlations were calculated between EEG data (dPAC, AAC) and self-reported characteristics (LSAS screening and testing, post-threat negative rumination, state nervousness, state approach motivation) within both the LSA and the HSA groups. Correlations between LSAS (testing) and EEG data (dPAC, AAC) during all conditions within-groups were not significant (all *p*s > .87, FDR-corrected). State nervousness and approach motivation before resting state and EEG data during resting state were not correlated (all *p*s > .87, FDR-corrected). Correlations for state nervousness and approach motivation before anticipation and EEG data during early anticipation showed a significant correlation between state nervousness and dPAC for LSA participants, *rho* = .632, FDR-corrected *p* = .007 (all other *p*s > .87, FDR-corrected). This correlation is displayed in Fig. 5. State nervousness and approach motivation after anticipation and EEG data during late anticipation were not correlated (all *p*s > .51, FDR-corrected). State nervousness and approach motivation before recovery, post-threat negative rumination and EEG data during early recovery were not correlated (all *p*s > .91, FDR-corrected). State nervousness and approach motivation after recovery, post-threat negative rumination and EEG data during late recovery were not significantly correlated (all *p*s > .87, FDR-corrected).

## Discussion

This study set out to investigate the utility of frontal delta-beta PAC and AAC (both an index of cross-frequency coupling; CFC) as neural indices of stress regulation in high versus low socially anxious females in an SPT. Two competing hypotheses were tested regarding the functional significance of delta-beta CFC in social anxiety. Based on the notion that frontal delta-beta CFC would reflect attentional control (and reduced trait anxiety), it was predicted that delta-beta PAC and AAC would be reflective of stress regulation efficiency, and thus be higher in LSA than in HSA participants during an SPT. In contrast, a competing account suggests that increased delta-beta CFC is indicative of maladaptive stress regulation in social anxiety, and thus higher delta-beta PAC and AAC would be expected in HSA relative to LSA females during an SPT. The current frontal delta-beta PAC findings speak to the suggestion that PAC was not related to trait social anxiety, since it was significant in both groups during all conditions, while no significant group differences were observed. However, PAC during early anticipation was positively correlated with state nervousness before anticipation in LSA participants, suggesting a relationship with state anxiety. Regarding frontal delta-beta AAC, our results seem to suggest that AAC is an index of stress regulation efficiency, since AAC was higher in LSA participants than HSA participants during the early anticipation condition. Moreover, significant delta-beta AAC was observed in the LSA group during resting state and all SPT conditions, while the HSA group showed no significant delta-beta AAC during any conditions.

The current results indicate that frontal delta-beta PAC was highly significant in both LSA and HSA participants during all conditions. Since delta-beta CFC is commonly interpreted to reflect subcortical-cortical crosstalk (Schutter et al., 2006), and is also present during efficient emotion regulation (Banks et al., 2007), one could suggest that both LSA and HSA participants efficiently regulated their anxiety during all conditions. However, there were no significant group differences in delta-beta PAC between LSA and HSA participants, while self-reported state and trait social anxiety did differ between groups. An alternative explanation of these significant delta-beta PAC findings might be due to a methodological issue, as illustrated by the simulation analyses. The same PAC analysis script was run on simulated data to test the analysis script, with simulations either containing strong CFC or no CFC (see Supplementary Materials for details). Unexpectedly, the simulated data both with and without CFC showed highly significant PAC, indicating that the PAC analysis is so sensitive with the current approach that it will always significantly differ from zero, even when there is no coupling present. However, the simulated data did show significantly more PAC in the data with introduced coupling as compared to the data without introduced coupling. Therefore, directly comparing groups / conditions on PAC seems to be more informative, as it can accurately distinguish between PAC being present or not. Since no significant differences were found in the current study between LSA and HSA participants, we can conclude that there were no group differences in PAC. As such, the current data cannot distinguish between the competing hypotheses of whether frontal delta-beta PAC reflects adaptive or maladaptive stress regulation during anxiety.

Importantly, frontal delta-beta PAC was found to be correlated with state nervousness during a stressful situation, but only in LSA participants, indicating that PAC seems to be sensitive to mild state anxiety. Although the current PAC findings fit nicely with the positive association between frontal delta-beta PAC and state anxiety as reported by Knyazev (2011), an important difference between our study and the Knyazev (2011) study is the group design. Here we used a between-subjects design, comparing high versus low socially anxious females. This design – together with the state nervousness and approach motivation self-reports – allowed us to better test whether delta-beta CFC would reflect a maladaptive stress regulation mechanism (CFC is higher in HSA) or an adaptive stress regulation mechanism (CFC is higher in LSA). In studies reporting only correlations with state anxiety (e.g., Knyazev, 2011) it is less clear whether the delta-beta CFC association with state anxiety is a normal or abnormal stress regulation response. The current findings seem to suggest that increased frontal delta-beta PAC reflects mildly increased state anxiety in moderately stressful situations, but does not differentiate adaptive from maladaptive stress regulation and thus is not sensitive to trait (social) anxiety. It should be noted, however, that only a few studies exist that have examined PAC to test affective neuroscientific hypotheses. Thus, there is a need for future work that will examine the validity of delta-beta PAC as being sensitive to a mild state of anxiety.

In contrast with PAC, frontal delta-beta AAC did show differences between groups in the current study. The observation that delta-beta AAC is significant in LSA only, and is significantly higher in LSA than in HSA participants during the early anticipation of an SPT, suggests that this cross-frequency coupling metric might indeed reflect a stress regulatory mechanism and is increased with lower (situation-specific) trait social anxiety. This is in line with previous associations of delta-beta AAC with increased attentional control (Morillas-Romero et al., 2015; Putman et al., 2012) and with reduced trait anxiety (Putman, 2011), but contradicts previous associations with increased social anxiety (Harrewijn et al., 2016; Harrewijn et al., 2017; Miskovic et al., 2010), and state anxiety (Knyazev, Schutter, & van Honk, 2006; Knyazev, 2011). It should be noted, however, that here we have used a different method to calculate the delta-beta AAC, which hampers a direct comparison between the current data and those reported in the literature. We opted to use within-subject analyses and calculated per participant and condition the correlation between delta and beta amplitude time series (with each epoch separately filtered for delta and beta frequencies). This approach was chosen in order to be able to compare current PAC and AAC results and to use a more reliable method of investigating the within-subject interaction between neuronal oscillations. This method differs, however, from the more commonly used Fast-Fourier transformation performed on single epochs to obtain delta and beta power that is then correlated between subjects. Despite these differences in analyses, the current findings corroborate the validity of using frontal delta-beta AAC to investigate the neural correlates of social anxiety and emphasize its association with adaptive stress regulation. To further explore CFC in affective processes, future research should consider calculating PAC and AAC within and between different brain locations and hemispheres, to prove that bilateral frontal regions indeed show the largest effect, and also to employ source modelling to clarify its sources. For example, some studies have compared delta-beta AAC within frontal and parietal regions and have only shown effects for parietal regions (Morillas-Romero et al., 2015), or have compared frontal delta-beta AAC between hemispheres and only found results within the right hemisphere (Miskovic et al., 2010). Moreover, frequency-frequency co-modulation plots can be used to explore other frequency bands besides delta-beta, to examine those frequency modulations that correlate most strongly with anxiety status. For example, there are some indications that delta-alpha AAC is sensitive to extraversion and behavioral inhibition (Schutter & Knyazev, 2012), and it would be interesting to study this further by comparing AAC with PAC. Most importantly, there is a great need for more research into the neural substrates that govern frontal delta-beta CFC, especially since the mechanistic and functional interpretation of AAC is unclear (Canolty & Knight, 2010).

Future work could also consider the following limitations of the present study. First, the resting state was recorded with eyes closed, while the anticipation and recovery conditions were recorded with eyes open. As this changes the topography and mean power of both delta and beta bands (Barry, Clarke, Johnstone, Magee, & Rushby, 2007), the resting state is not directly comparable to the SPT conditions. Second, the current data are restricted to the female gender. We only included female participants, since previous studies have shown evidence of gender differences in the prevalence and severity of internalizing psychopathology, such as social anxiety disorder, as well as in emotion regulation strategies and its neural mechanisms (Kudielka & Kirschbaum, 2005; Nolen-Hoeksema, 2012; Seo, Ahluwalia, Potenza, & Sinha, 2017; Turk et al., 1998). To increase our understanding of the functional significance of delta-beta CFC, it is of critical importance to examine its behavior during a social stressor in both genders. Third, to generalize these findings to social anxiety disorder (or other internalizing disorders), future work should examine delta-beta CFC in individuals with a psychiatric diagnosis, since the current findings are based on female undergraduates with subclinical social anxiety levels.

To conclude, the current study was the first to investigate frontal delta-beta AAC and PAC as a measure of stress regulation in high and low socially anxious females using a social performance task. Within-subject delta-beta PAC and AAC were calculated during resting state, anticipation, and recovery conditions. Results showed that AAC distinguished low and high social anxiety during the anticipation of an SPT and was significant during all conditions in LSA participants only. This indicates that frontal delta-beta AAC is sensitive to trait anxiety and reflects an adaptive stress regulation mechanism. PAC did not show any group differences, and instead was shown to be correlated with state anxiety during the anticipation of an SPT in LSA participants only. This suggests that frontal delta-beta PAC is sensitive to mild state anxiety in low socially anxious participants but cannot distinguish between adaptive or maladaptive stress regulation. There is, however, a need for future research to unravel the functional mechanism of frontal delta-beta AAC and PAC.

## Electronic supplementary material

ESM 1 (DOCX 426 kb)

## Footnotes

1 The criterion of right-handedness was of relevance to the social evaluation paradigm (involving motor responses) administered in the research protocol (reported elsewhere).

2 Six clean epochs were chosen for every participant to ensure an equal number of epochs for every participant and group, since differences in epochs between participants/conditions negatively impact the signal-to-noise ratio (Cohen et al., 2009). Clean epochs were randomly chosen (spread evenly) out of the available clean epochs (maximum ten). Participants with less than six artefact-free epochs were excluded from analyses (HSA: final N = 20, with 4 excluded; LSA: final N = 32, with 2 excluded). Although more and longer epochs are recommended to increase signal-to-noise ratio and reduce false positives (Berman et al., 2015), having more and shorter epochs negatively influences phase stability (Nunez & Srinivasan, 2006). However, increasing the number of epochs by increasing the length of the SPT will reduce stress induction effects. Therefore, the chosen number of epochs and their length are based on a trade-off between multiple factors.

3 Down-sampling to 128 Hz was performed for every epoch in order to design more precise filters (Alessio, 2016). This reduces phase slips – spurious discontinuities in the analytic phase caused by interference from components with different frequencies (Freeman, 2007) – thereby stabilizing the delta phase.

4 Simulated data were analyzed to assure proper functioning of the dPAC and AAC analysis script and to give a reference frame for the minimum and maximum to-be-expected values. Further details of the simulation and its results can be found in the Supplementary Materials.
