# Supplementary material for: Frontal delta-beta cross-frequency coupling in high and low social anxiety: An index of stress regulation?
Source: Cogn Affect Behav Neurosci. 2018 May 17;18(4):764–77. doi: 10.3758/s13415-018-0603-7 (PMC6096649; doi:10.3758/s13415-018-0603-7)

**Supplementary materials**

*1. Control analyses of dPAC and AAC*

New data were simulated and analyzed to assure proper functioning of the dPAC and AAC analysis script and to give a reference frame for the minimum and maximum to-be-expected values. Using these control analyses, we can be sure that this specific script can distinguish between data where we know there is strong coupling present vs. no coupling present. The Matlab (MATLAB 2017a) script to simulate the data was based on the description of simulated data by van Driel et al. (2015); and is freely available online (https://github.com/ESPoppelaars/Cross-frequency-coupling).

The simulated delta signal was a series of exponents to represent a phase-biased cosine (with g = .05):

$$S(t;c)=\sum_{c}^{T} e^{-(t-c)^{2}/2g^{2}}$$

The delta signal was non-sinusoidal in order to simulate the bias of a non-uniform phase-angle distribution. Each epoch of the delta signal was set to a random frequency between 1 and 4 Hz, with an amplitude randomly fluctuating between 1 and 4 Hz in case of introduced coupling, and between 1 and 50 Hz in case of no introduced coupling. The simulated beta signal was a cosine with a random frequency between 14 and 30 Hz for each epoch, characterized by either a strong coupling with delta (both phase and amplitude), or no coupling. Moderate (k = 6) or strong (k = 20) pink noise was superimposed on the combined delta and beta signal to represent noise, such as in a standard EEG signal. The same characteristics as the current input data were used: six epochs of eight seconds, a sampling rate of 512 Hz, and a composite measure of three electrodes. A generated sample size of N = 20 was used, such as in the smallest group of our data (HSA). Finally, the same dPAC script was run as described in sections 2.7 -2.8 of the manuscript.

Results showed that in the simulated data with introduced coupling and moderate noise, delta-beta dPAC differed significantly from zero: magnitude = 0.045, *Z* = 7.41, *t*(19) = 51.16, *p* < .001; as did delta-beta AAC: *r* = .12, *t*(19) = 16.03, *p* < .001. In the simulated data with no introduced coupling, results showed that dPAC still differed significantly from zero: magnitude = 0.0056, *Z* = .82, *t*(19) = 10.56, *p* < .001; although AAC did not: *r* = .005, *t*(19) = 1.54, *p* = .14. However, both dPAC and AAC showed significant differences between the data with and without coupling present; dPAC: *t*(19) = 37.69, *p* < .001; AAC: *t*(19) = 14.51, *p* < .001.

Similarly, results showed that in the simulated data with introduced coupling and strong noise, results showed that delta-beta dPAC differed significantly from zero: magnitude = 0.021, *Z* = 1.50, *t*(19) = 9.99, *p* < .001; as did delta-beta AAC: *r* = .014, *t*(19) = 3.32, *p* = .004. In the simulated data with known no coupling present, results showed that dPAC still differed significantly from zero: magnitude = 0.014, *Z* = .90, *t*(19) = 12.98, *p* < .001; although AAC did not: *r* = -.004, *t*(19) = 1.23, *p* = .233. Again, both dPAC and AAC showed significant differences between the data with and without coupling present; dPAC: *t*(19) = 3.32, *p* = .004; AAC: *t*(19) = 3.25, *p* = .004.

These values verify that the script can accurately distinguish between data with and without coupling present, while also providing a reference frame for the minimum and maximum values that can be expected in the current data. However, the significant dPAC values in the simulated data with no introduced coupling is worrisome and needs to be kept in mind. Polar plots of dPAC values are displayed in Figures S1A and S1B.

*2. Down sampling*

Figures S2A and S2B show the effect of down sampling from a sampling rate of 512 Hz to 128 Hz on the stability of the Hilbert-transformed delta phase.

**References**

MATLAB and Signal Processing Toolbox Release 2017a. (n.d.). Natick, Massachusetts, United States: The MathWorks, Inc.

van Driel, J., Cox, R., & Cohen, M. X. (2015). Phase-clustering bias in phase–amplitude cross-frequency coupling and its removal. *Journal of Neuroscience Methods*, *254*, 60–72. http://doi.org/10.1016/j.jneumeth.2015.07.014

**Supplementary Figures**

Figure S1.

Polar plots of dPAC for data with moderate noise and A) with introduced coupling, and B) without introduced coupling.


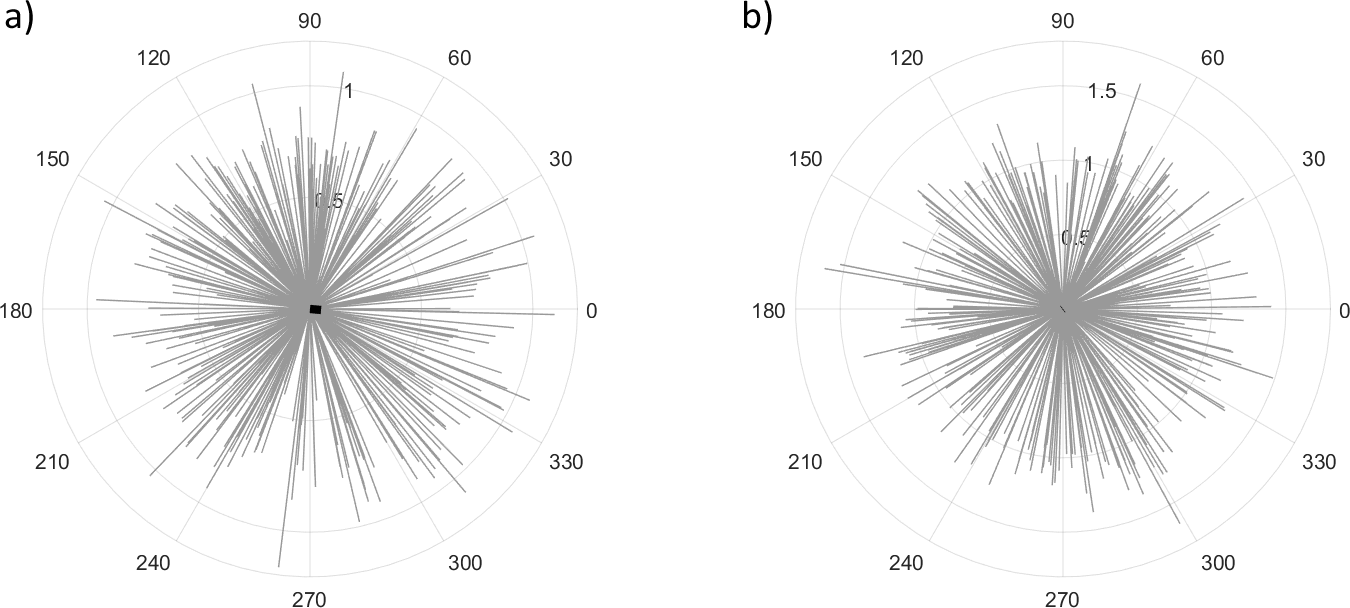


Figure S2

Example plots of the delta phase for epochs (during the early anticipation of an LSA participant) with a sampling rate of A) 512 Hz, and B) 128 Hz.


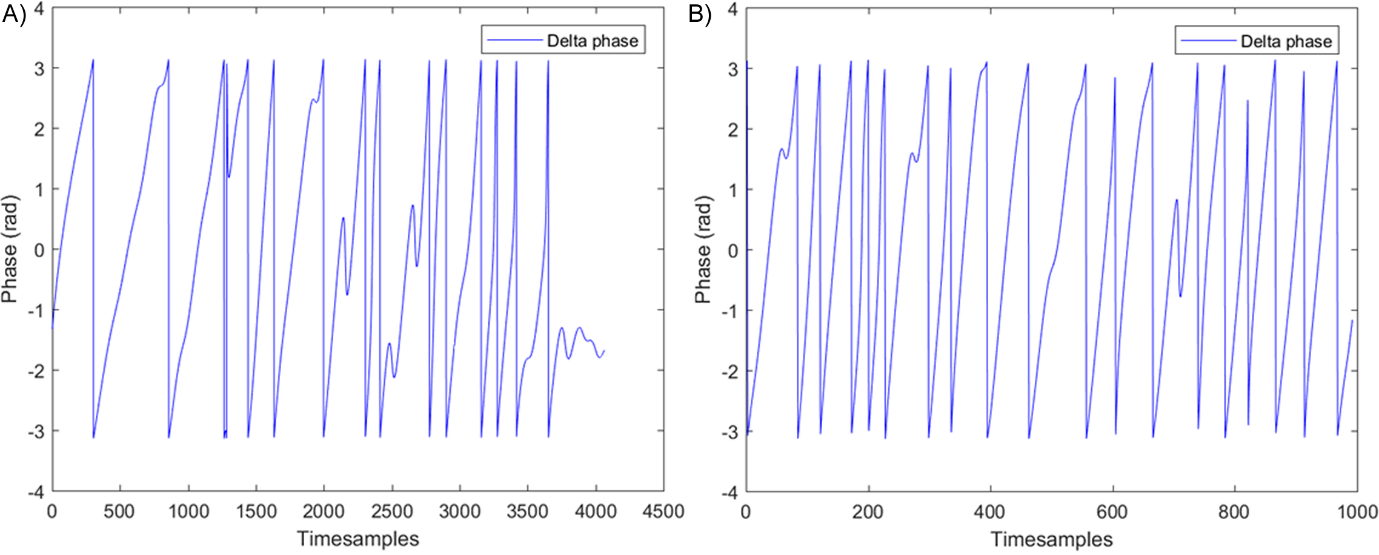

Supplement: Supplementary file 1 — (DOCX 426 kb) [file 13415_2018_603_MOESM1_ESM.docx]
